# Characterization of nuclear DNA diversity in an individual *Leymus chinensis*

**DOI:** 10.3389/fpls.2023.1157145

**Published:** 2023-06-06

**Authors:** Haoyang Yu, Lijuan Ma, Ye Zhao, Gaowa Naren, Haiyan Wu, Yongwei Sun, Lei Wu, Lingang Zhang

**Affiliations:** School of Life Sciences, Inner Mongolia University/Key Laboratory of Herbage and Endemic Crop Biotechnology, Ministry of Education, Hohhot, Inner Mongolia, China

**Keywords:** intraorganismal genetic heterogeneity, somatic mutation, SNP, evolution, *Leymus chinensis*

## Abstract

Intraorganismal genetic heterogeneity (IGH) exists when an individual organism harbors more than one genotype among its cells. In general, intercellular DNA diversity occurs at a very low frequency and cannot be directly detected by DNA sequencing from bulk tissue. In this study, based on Sanger and high-throughput sequencing, different species, different organs, different DNA segments and a single cell were employed to characterize nucleotide mutations in Leymus chinensis. The results demonstrated that 1) the nuclear DNA showed excessive genetic heterogeneity among cells of an individual leaf or seed but the chloroplast genes remained consistent; 2) a high density of SNPs was found in the variants of the unique DNA sequence, and the similar SNP profile shared between the leaf and seed suggested that nucleotide mutation followed a certain rule and was not random; and 3) the mutation rate decreased from the genomic DNA sequence to the corresponding protein sequence. Our results suggested that Leymus chinensis seemed to consist of a collection of cells with different genetic backgrounds.

## Introduction

The definition of an individual organism, traditionally defined by the invariable presence of a physiological unit and genetic homogeneity, is challenged by situations such as intraorganismal genetic heterogeneity (IGH), which refers to the presence of more than one genotype in a single organism (Schweinsberg et al., 2015). Cells within a multicellular individual are derived from a common single-celled ancestor and are closely related with little expected mutation. However, mosaicism can still be detected, albeit at a very low frequency (Sutherland and Watkinson, 1986; Schoen and Schultz, 2019). Whole-genome mutation rates are always estimated *via* mutation accumulation studies and phenotypic progeny assays together with quantitative genetic models. These rates generally range between 0.1 and 1.0 per genome per generation (Roles and Conner, 2008; Kondrashov and Kondrashov, 2010; Lovell et al., 2017; Exposito-Alonso et al., 2018; Orr et al., 2020). *Arabidopsis thaliana* has a per-generation mutation rate of 7.1× 10^-9^ mutations per base (Ossowski et al., 2010). Approximately 40 nucleotide differences were identified between two branch tips of a single 234-year-old oak, suggesting a somatic mutation rate of 4.2-5.2×10^-8^ substitutions/site/generation (Schmid-Siegert et al., 2017). A somatic base substitution rate of 2.7×10^-8^ per base pair was estimated within a single generation of Sitka spruce, which is regarded as one of the highest estimated per-generation rates of mutation among eukaryotes (Hanlon et al., 2019). In some species, the rate of mutation accumulation per unit time in the shoot apical meristem is lower than that in root apical tissues (Wang et al., 2019). All of these somatic mutations are obviously maintained at a very low level and are difficult to directly detect through more accurate methods, such as sequencing from bulk tissue.

Genetic mosaics are generated by a number of mechanisms. During cell mitosis in multicellular organisms, DNA integrity is disturbed by multiple potential replication errors and DNA damage caused by environmental mutagens from which plants cannot escape, such as solar UV-B light (Yao and Kovalchuk, 2011; Jiang et al., 2014), temperature stress (Saini et al., 2017) and genotoxic chemicals (Lavigne et al., 2017), or by endogenous DNA-damaging oxyradicals (Hu et al., 2016). These kinds of DNA variations occur in a passive and random way.

IGH has long been seen as a potential threat to solitary organisms, as it could lead to antagonistic interactions among different genotypes, as observed in the development of tumors and autoimmune diseases (Pineda‐Krch and Lehtila, 2004). In some extreme cases, IGH can cause the death of one or more genotypes or even the whole organism (Hoffman, 2004). Until recently, viable IGH was considered to represent an exceptional situation. It presents some benefits (Ben‐Shlomo, 2017; Our et al., 2020), such as increasing the phenotypic plasticity (Medina et al., 2015), competitive abilities (Ballarin et al., 2015; Forsman et al., 2015) and/or fitness of the organism (Folse and Roughgarden, 2012; Simberloff and Leppanen, 2019). Whether IGH is neutral, beneficial, or deleterious can vary across the different species and is closely related to environmental quality.

In this study, somatic mutation of nuclear genes was detected through DNA sequencing of an individual leaf and seed of *Leymus chinensis*, a perennial forage grass that exhibits abiotic stress tolerance and has high nutritional value (Jin et al., 2008; Zhai et al., 2014). The intercellular nucleotides varied and contained single nucleotide polymorphisms (SNPs).

## Materials and methods

### Plant materials and growth conditions

Mature seeds of two ecotypes of *Leymus chinensis*, grey green (GG) and yellow green (YG), were collected from the Huhetala grassland in Inner Mongolia, China (111°37′E, 40°50′N, 1040 m asl), in late July 2020. The seeds of *Leymus multicaulis* were presented by US National Plant Germplasm System (NPGS). *Leymus* seeds, together with the seeds of rice (*Oryza sativa L. japonica*) and wheat (*Triticum aestivum L.*), were surface sterilized by soaking in 45% sodium hypochlorite (NaClO) for 15 min with constant shaking. Then, the seeds were rinsed 5 times with sterile distilled water to remove residual NaClO. The seeds were vernalized at 4°C for 3 days and then sown onto 0.7% Murashige and Skoog (MS) agar plates supplemented with 1% (w/v) sucrose. The plants were maintained under 12 h light/12 h dark cycles (approximately 70 μmol m^-2^s^-1^ light) at a constant temperature of 22°C. After 2 weeks of growth on MS medium, the seedlings were transferred to soil for growth for another 2 weeks, and then one piece of a fully expanded leaf sample was used for DNA extraction. For seed DNA purification, a single *Leymus* seed was randomly selected from the mature seed collection for the extraction of total genomic DNA after removing its glume. Wild-type *Arabidopsis thaliana* (Column-0) was used as a control for all experimental procedures performed in *Leymus chinensis*. The seed sterilization and seedling growth procedures were the same for both species, except that 20% NaClO was used for *Arabidopsis* seed surface sterilization. A single leaf of an *Arabidopsis* seedling was randomly chosen for DNA isolation.

### DNA extraction and gene cloning

**Figure 1:**
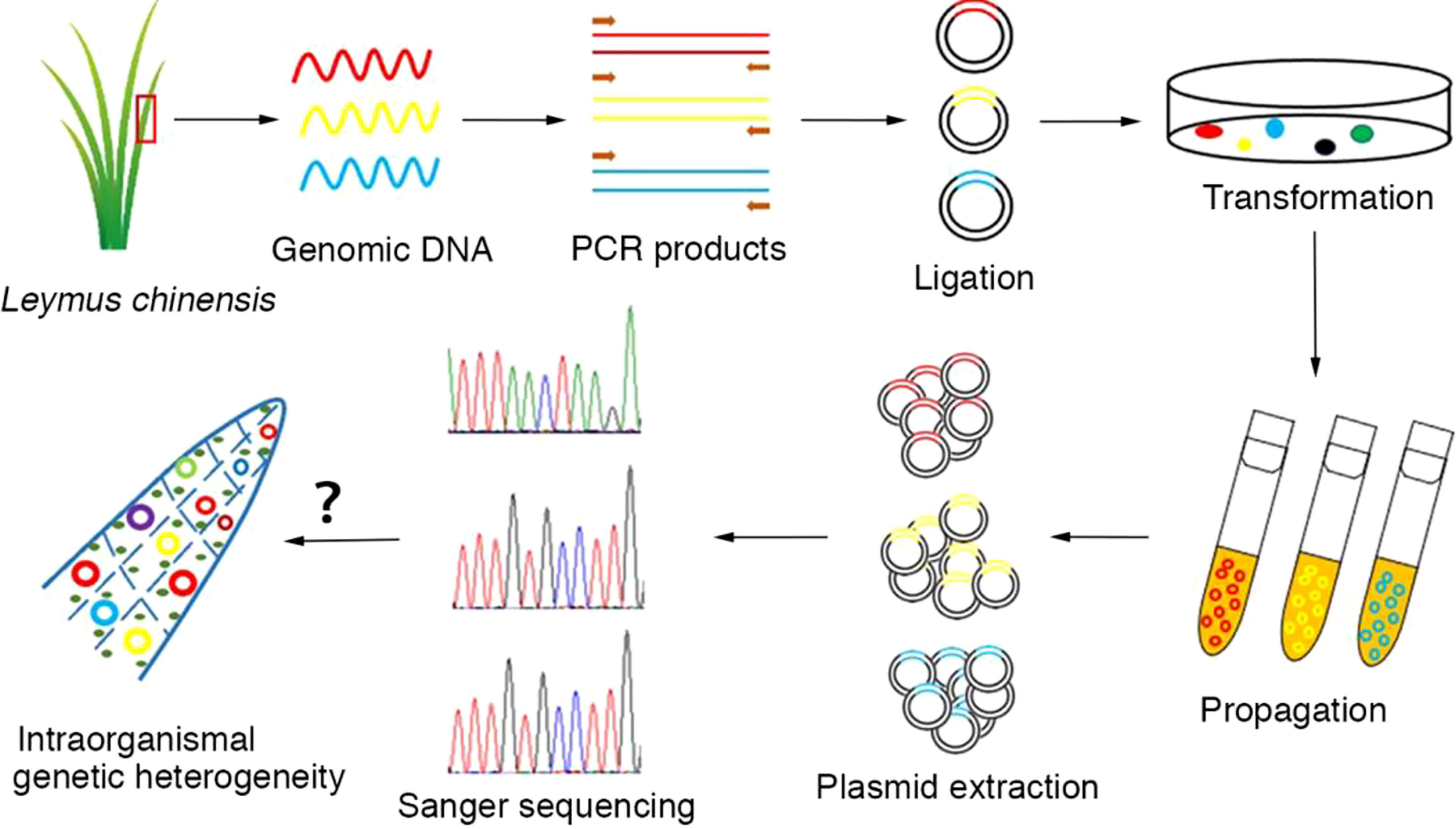
Workflow for the analysis of intraorganismal genetic heterogeneity in the *Leymus* leaf. An individual leaf of *Leymus chinensis* was processed to isolate genomic DNA, which was then used as a template for target DNA (nuclear genes: *VIPP1* and *XLG3*; chloroplast genes: *psbA* and *ndhH*) cloning with high-fidelity DNA polymerase (KOD-plus). The PCR products were inserted into plasmids. The positive clones identified by colony PCR and propagated in *E. coli* (DH5α). The recombinant plasmids containing DNA inserts were extracted and used for the sequencing of target genes. In general, more than 50 independent recombinant plasmids were selected for the sequencing of each gene/DNA fragment with the Sanger method.

Total genomic DNA was extracted from plant tissues using a PlantZol Kit (TransGen Biotech, China). The plant tissue (leaf or seed) was ground to a fine powder in liquid nitrogen. DNA was released *via* PlantZol reagents. After extraction with an equal volume of phenol−chloroform solution, genomic DNA was precipitated from the supernatant with isopropanol. The genomic DNA pellets were dissolved in distilled water after washing with 70% alcohol and stored at -20°C. Because there is no information available on the genomic sequence of *Leymus chinensis*, homologous genes in Aegilops *tauschii* (http://plants.ensembl.org/Aegilops_tauschii/Info/Index?db=core) were used to design primers for cloning the target genes in this study. Primer sequences are listed in **Table S2**. PCR amplification was carried out with KOD-plus polymerase (Toyobo, Japan) following the manufacturer’s instructions in an Eppendorf thermocycler (Eppendorf, Germany). The target DNA sequences were amplified in a 50 μl standard reaction system containing 30 ng of genomic DNA, 1.0 mM Mg^2+^, 0.6 μM 3’-and 5’-end primers, 0.2 mM nucleotides, 1× PCR buffer, and 1.0 unit of KOD-plus polymerase. The PCR cycling conditions were as follows: 2.0 min of predenaturation at 94°C followed by 30 cycles of 15 sec of denaturation at 94°C, 30 sec of annealing at the specific temperature corresponding to the primer set and extension at 68°C. The product yields were analyzed on 1.0% agarose gels stained with Goldview nucleic acid dye. A single amplicon of the correct size was selected and cloned into a pEASY^®^-Blunt Zero vector (TransGen Biotech, China). Positive clones identified by colony PCR could be picked directly from the corresponding numbered field on the selective plate. The recombinant plasmids containing DNA inserts were extracted and used for the sequencing of target genes. In general, more than 50 independent recombinant plasmids were used for the sequencing of each gene/DNA fragment with the Sanger method (Sangon Biotech, China). The experimental process is outlined in **Figure 1**.

### Single-cell isolation

Fully expanded leaves of *Leymus chinensis* were excised and submerged gently in enzyme solution (2.0% [w/v] cellulase R10, 0.6% [w/v] macerozyme R10, 0.4 M mannitol, 20 mM KCl, 10 mM CaCl_2_, 0.1% BSA and 20 mM MES, pH 5.7). After incubation at room temperature for 3 h, protoplasts were collected by centrifugation at 100 × g for 2 min and washed with ice-cold wash buffer (154 mM NaCl, 125 mM CaCl_2_, 5 mM KCl and 2 mM MES, pH 5.7) 3 times. The protoplasts were diluted with wash buffer to a final density of 1000 protoplasts/ml. The protoplast suspension was fractioned with a 0.1-10 μl micropipette (Eppendorf, Germany) into a 0.5 μl drop and checked under a microscope. A drop containing a single intact protoplast was used to extract and amplify whole genomic DNA.

### Whole genome amplification of a single cell

The isolated *Leymus* protoplasts were subjected to the REPLI-g Single Cell Kit (Qiagen, Germany) following the manufacturer’s protocol. Briefly, 4.0 μl cell suspension with 3.5 µl PBS (supplied by the kit) added to the cell drop solution was incubated with 3 μl Buffer D2 for 10 min at 65°C. After stopping the reaction by adding 3 μl Stop Solution, a master mix comprising 9 μl H_2_O, 29 μl REPLI-g Reaction Buffer and 2 μl REPLI-g DNA Polymerase was added. The reaction was performed for 8 h at 30°C. The WGA amplification was stopped by inactivating REPLI-g DNA Polymerase at 65°C for 3 min. The amplified DNA was diluted 1:100, and 2 μl of diluted DNA was used for subsequent PCR.

### Nuclear visualization and chromosome staining

To monitor the nuclear size in somatic tissue, small pieces of mature leaves were fixed in freshly prepared 3:1 ethanol-acetic acid fixative for at least 1 h and then cleared in 70% ethanol. The leaf pieces were incubated for 30 min in PBST (137 mM NaCl, 2.7 mM KCl, 4.3 mM Na_2_HPO4, 1.4 mM KH_2_PO4, and 0.05% Triton X-100, pH 7.2) containing 1.0 μg/ml 4′,6-diamidino-2-phenylindole (DAPI) at room temperature and then washed three times with PBST. The DAPI-stained nuclei were photographed by a Nikon Eclipse Ci-S fluorescence microscope (Nikon, Japan), and the projection area of each nucleus was measured using ImageJ software. For chromosome counting of *Leymus chinensis*, root tips 0.5-1.0 cm in length were excised and treated with 0.04% 8-hydroxyquinoleine to increase the number of metaphase cells and chromosome condensation. Following the same method of fixation and DAPI staining as that used for leaves, the root tips were squashed on slides. The condensed chromosomes were captured by a Nikon Eclipse Ci-S fluorescence microscope (Nikon, Japan).

### Flow cytometry

The mature leaves were chopped with a sharp razor blade in 400 μl of CyStain UV Precise P nuclei extraction buffer and mixed with 1600 μl of CyStain UV Precise P staining buffer (both Partec) containing 1 mg/ml DAPI. Released nuclei were purified by sieving through 30 μm nylon mesh. Fluorescence of the nuclei was measured by a CyFlow^®^ Ploidy Analyzer (Partec, Germany).

### High-throughput sequencing and data quality control

Specific PCR products were sent to Sangon Biotech (Sangon, China) for library preparation and sequencing. Briefly, the PCR products were checked using electrophoresis in 1% (w/v) agarose gels in TBE buffer stained with SYBR Green I and visualized under UV light. After purification of the amplicon products, another PCR was set up as follows: DNA (10 ng/μl), 2 μl; universal P7 primer with index (10 μM), 1 μl; P5 primer with index (10 μM), 1 μl; and 2×PCR Ready Mix, 15 μl (total 30 μl) (Kapa Biosystems, USA). The plate was sealed, and PCR was performed in a thermal instrument (Bio-Rad, USA) using the following program: 1 cycle of denaturing at 98°C for 3 min, then 5 cycles of denaturing at 94°C for 30 s, annealing at 55°C for 20 sec, and elongation at 72°C for 30 s, and a final extension at 72°C for 5 min. Then, we used AMPure XP beads to purify the amplicon. The libraries were then quantified and pooled. Paired-end sequencing of the library was performed on MiSeq sequencers with a PE300 model (Illumina, USA).

Raw reads were filtered according to three steps: 1) adaptor sequences were removed by cutadapt; 2) low-quality bases were removed from 3’ to 5’ ends (Q<20) by PRINSEQ-lite; and 3) chimera sequences were removed by using arch software in *de novo* mode with the default parameters. The remaining clean data were used for further analysis. Briefly, a python script was used to select the target sequence when both the forward primer and reverse primer perfectly matched, and then the unique sequences and total sequences were calculated. After that, the unique sequence with a proportion over 1% (for tissue DNA) or the top 10 sequences (for single cell DNA) were used for multiplex alignment with ClustalW software.

### RNA isolation and cDNA synthesis

TRIzol reagent (Life Technologies, USA) was used for total RNA isolation according to the manufacturer’s instructions. Prior to cDNA synthesis, genomic DNA was removed by adding 1 unit of RNase-free DNase I and incubating the sample for 30 min at 37°C and then for 10 min at 65°C to inactivate the enzyme. For first-strand cDNA synthesis, 1 μg of total RNA was used as a template, and reverse transcription was performed using the RevertAi First Strand cDNA Synthesis Kit (Thermo Scientific, USA). Two microliters of the first strand cDNA synthesis reaction mixture was used as a template for subsequent PCR in a 50 μl total volume.

### Multiple sequence alignment analysis

The quality of the sequencing reads was evaluated according to chromatogram performance in MySequence software, with special attention given to low-frequency polymorphisms. The individual reads of each gene/haplotype were aligned according to either the DNA sequence or the corresponding translated amino acid sequence using ClustalW with the default parameters. Multiple alignments were visualized with Jalview to identify variant nucleotids or residues in the sequences. Conserved nucleotids or residues are highlighted based on the percent identity in the alignment (blue: >80% agreement, medium blue: >60% agreement, and light blue: >40% agreement; only the residues that agreed with the consensus nucleotide/residue for each column are colored). DNA sequence divergence was calculated *via* neighbor-joining or average distance. In the obtained tree, identical entries were clustered together as a group, and each cluster was assumed to represent a ‘haplotype’.

### Statistical analysis of nucleotide diversity

Nucleotide diversity analysis was performed using DnaSP v5.10 software. Mutations, including transitions and transversions, were counted according to the software setup. Nucleotide deletions/insertions were artificially modified on the basis of software calculations. An extended deletion at a given position was recorded as a single-nucleotide deletion. The total number of SNPs represented the summary of transitions, transversions and deletions/insertions. The haplotypes referred to here are the groups comprising identical reads among the total number of sequences. The exon/intron structures of *Leymus* genes were determined by referring to their orthologs in Aegilops *tauschii* (http://plants.ensembl.org/Aegilops_tauschii/Info/Index?db=core).

### Leaf anatomical observations

Leaf pieces from the middle of the leaf blade were fixed in FAA (5:5:90 5% formalin:5% glacial acetic acid:70% alcohol) solution for 24 h. After dehydration and immersion, the samples were embedded in pure paraffin. The paraffin blocks containing the samples were then sliced to a 5 μm thickness using a rotary microtome. Leaf sections were stained first with safranin and then with Alcian blue. Bright light images were acquired with a microscope (Nikon Eclipse E100, Nikon). For transmission electron microscopy (TEM), fixed *Leymus chinensis* leaf pieces were further dehydrated in a graded ethanol series (50, 70, 90, and 100%). Ethanol was subsequently replaced using a series of epoxy resin dilutions (50, 70, 90, and 100%). Then, the resin was hardened for 2 days at 60°C. Sections were stained with 2% lead citrate and examined using a transmission electron microscope (HT7800, Hitachi) at 80 kV.

## Results

### Heterozygosity of unique nuclear DNA sequences in an individual *Leymus chinensis* leaf

**Figure 2:**
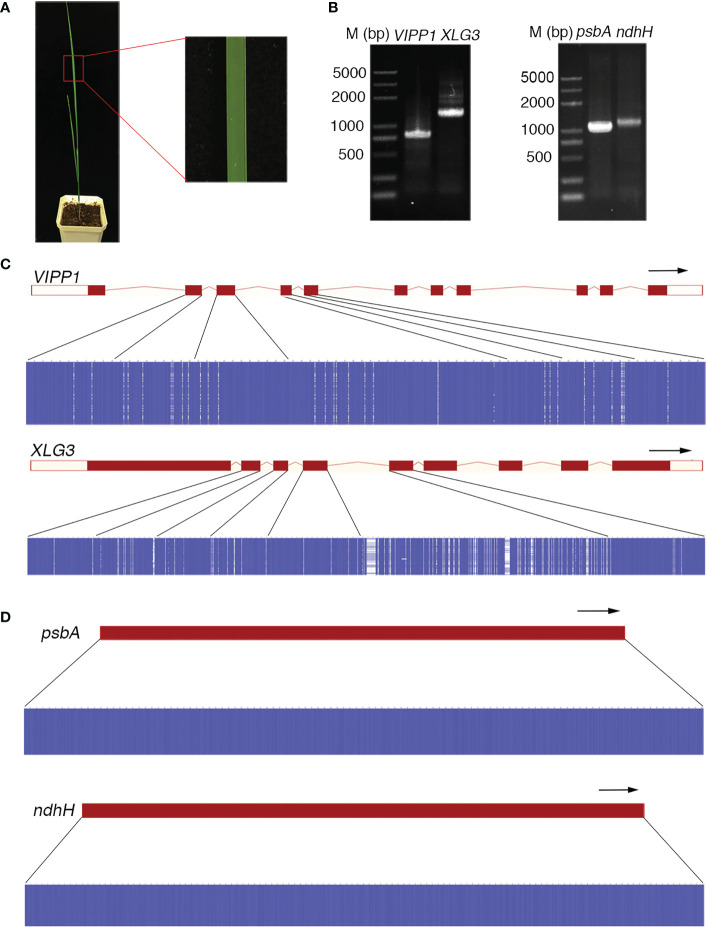
Nucleotide diversity of nuclear genes in an individual leaf of *Leymus chinensis*. **(A)** A four-week-old *Leymus chinensis* seedling and an enlarged image of a leaf fragment. **(B)** PCR products of *VIPP1*, *XLG3*, *psbA* and *ndhH*. **(C, D)** Multiple sequence alignments of sequenced reads of *VIPP1*, *XLG3*, *psbA* and *ndhH*. Nucleotides were colored for percent identity using Jalview. The transcript diagrams of different genes are above those of the corresponding DNA sequences. Both the exons and introns of the DNA sequences are marked according to the transcript diagram.

The DNA sequence often defines the attributes, nature and type of an individual and is commonly used as a marker to identify species. This application is based on two premises: 1) the DNA sequence should be distinct for different species, and 2) the DNA sequence of an individual should be consistent. However, the DNA sequences of *Leymus chinensis* do not follow this rule. In this study, a high frequency of SNPs was found in nuclear genes (such as *VIPP1* and *XLG3*) in an individual leaf of *Leymus chinensis*, while the integrity of chloroplast genes (such as *psbA* and *ndhH*) was maintained. Single bands of genomic fragments and chloroplast genes could be obtained with high-fidelity polymerase from the total genome extracted from a piece of *Leymus chinensis* leaf (**Figures 1**, **2A, B**). Fifty individual sequencing reads of each gene/fragment integrated into a plasmid were aligned separately. The DNA fragments of *VIPP1* and *XLG3* showed excessive polymorphism, not only in introns but also in exons (**Figure 2C**; **Supplementary Table S1**). However, nucleotide variation was not detected in the *psbA* and *ndhH* chloroplast genes (**Figure 2D**). The representative chromatogram of DNA sequences clearly showed a definite nucleotide signal peak, which excluded the possibility of nucleotide mutation induced by the sequencing method (**Supplementary Figure S1**). *Arabidopsis thaliana* was chosen as a control for *Leymus chinensis* throughout the process from genome extraction to gene sequencing. No mutations were found in *VIPP1* and *psbA* in *Arabidopsis* (**Supplementary Figure S2**), which suggested that the identified intraorganismal nucleotide polymorphism was specific to *Leymus chinensis*. High mutation rates of 39.3 (for *VIPP1*) and 80.0 (for *XLG3*) SNPs per kilobase pair were detected, which were far greater than the rates reported in other species (Schoen and Schultz, 2019). There were more mutations in introns than in exons for both DNA fragments (**Supplementary Table 1S**). Among 50 individual reads, 15 and 18 haplotypes were found for *VIPP1* and *XLG3*, respectively (**Supplementary Figure S3, Supplementary Table S1**). All of the above data clearly demonstrated that nuclear DNA sequences from an individual *Leymus chinensis* leaf were heterozygous.

### High levels of nuclear DNA polymorphism in an individual *Leymus chinensis* leaf were confirmed by high-throughput sequencing

**Figure 3:**
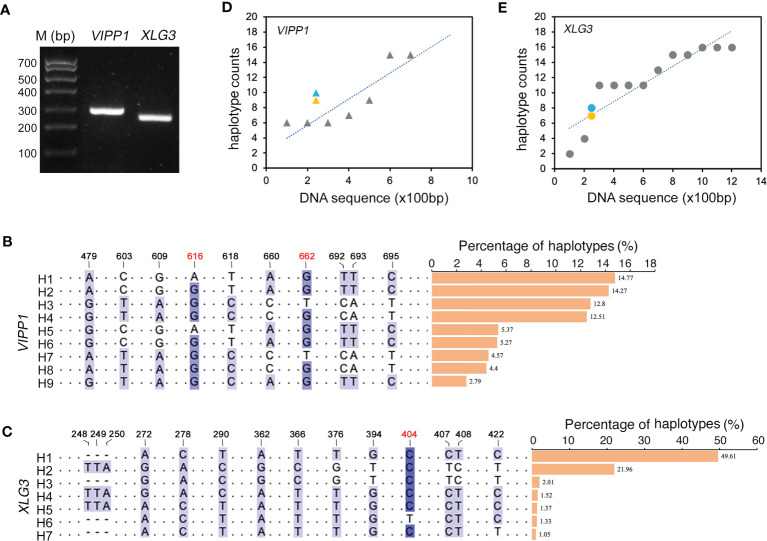
Nucleotide diversity of *VIPP1* and *XLG3* affirmed by high-throughput sequencing. **(A)** PCR products of shorter *VIPP1* and *XLG3*. The SNP sites of aligned *VIPP1* **(B)** and *XLG3* **(C)** sequences. Each unique sequence represents one haplotype that is marked with “H” at the beginning of the sequence. The number above indicates the site of SNPs in the corresponding longer *VIPP1* or *XLG3* fragments in **Figure 2**. The new SNP sites are marked with red figures. The proportions of each haplotype are represented by a histogram. **(D, E)** The correlation between haplotypes and DNA length of *Leymus* nuclear genes. Orange marks indicate the haplotype number of *VIPP1* (triangle) and *XLG3* (round dot) sequenced with NGS, while the haplotypes of their corresponding fragments in longer *VIPP1* and *XLG3* are marked in blue.

Compared to the traditional Sanger sequencing method, high-throughput sequencing (also called next-generation sequencing, NGS) can enormously increase the number of reads, achieving a much greater depth of coverage. Because NGS could accurately generate read lengths of approximately 300 bp, shorter *VIPP1* (~293 bp) and *XLG3* (~259 bp) segments truncated from their corresponding longer sequences in **Figure 2C** were selected for sequencing with NGS (**Figure 3A**, **Supplementary Figure 4S**). After raw read filtering, the clean reads (74,683 for *VIPP1* and 110,169 for *XLG3*) were used for frequency counts of unique haplotypes. **Figures 3B, C** show identical (black Figures) and new (red Figures) SNPs compared with their corresponding sequences generated by the Sanger method. In addition, some SNPs from the longer *VIPP1* and *XLG3* sequences were absent in the shorter ones generated by NGS (**Supplementary Figure S4**, black arrows). Nevertheless, the majority of SNPs were shared by two kinds of DNA fragments sequenced by the Sanger and NGS methods for both genes. Thus, a similar pattern of SNPs resulting from NGS further confirmed the nucleotide diversity of *Leymus chinensis*. Regardless of whether the Sanger method (green) or NGS (orange) were used, the same part of the DNA sequence formed a similar number of haplotypes for *VIPP1* and *XLG3*. However, as the DNA sequence was extended, the number of haplotypes increased for both *VIPP1* and *XLG3* fragments (**Figures 3D, E**).

To clarify whether the SNP haplotypes were a universal character for the *Leymus* genome sequence, three other DNA fragments (*ACT1*, *NYC1* and *VTE3*) were chosen for NGS. The unique sequences with a proportion over 1.0% were arranged from high to low and aligned using ClustalW software (**Supplementary Figures S5A, B**). The selected regions of three genes comprised one intron and two exons. **Figure S5C** illustrates that the SNP density in introns was higher than that in exons, similar to that in *VIPP1* and *VTE3* (**Figures 2C, D**; **Supplementary Table S1**). Different numbers of haplotypes were derived from these polymorphisms: 5 haplotypes for *ACT1*, 11 haplotypes for *NYC1* and 12 haplotypes for *VTE3*. The percentage of different haplotypes for these genes varied greatly (**Supplementary Figure S5D**). The DNA diversity of *ACT1*, *NYC1* and *VTE3* demonstrated that nucleotide mutations should be universal traits for nuclear DNA sequences in *Leymuschinensis*. In order to examine if the similar DNA diversity also occurred in other species of Leymus Hochst., the homologic genes of *ACT1*, *NYC1*, *VTE3*, *XLG3* and *VIPP1* of *Leymus multicaulis* were cloned and sequenced. Consistent with the results of *Leymus chinensis*, these genes of *Leymus multicaulis* also exhibited similar patterns of DNA polymorphisms (**Supplementary Figure S6**): numerous SNP sites were detected between different haplotypes of each gene, and SNP density in introns was also higher than that in exons; except of *ACT1* and *VIPP1*, different numbers of haplotypes with different percentages were produced in the other DNA fragments of *Leymus multicaulis*. The above results suggested that nuclear DNA polymorphism was not a unique feature of *Leymus chinensis*, it also occurred in its relative species, *Leymus multicaulis*.

### Nuclear size and chromosome ploidy of *Leymus chinensis*

**Figure 4:**
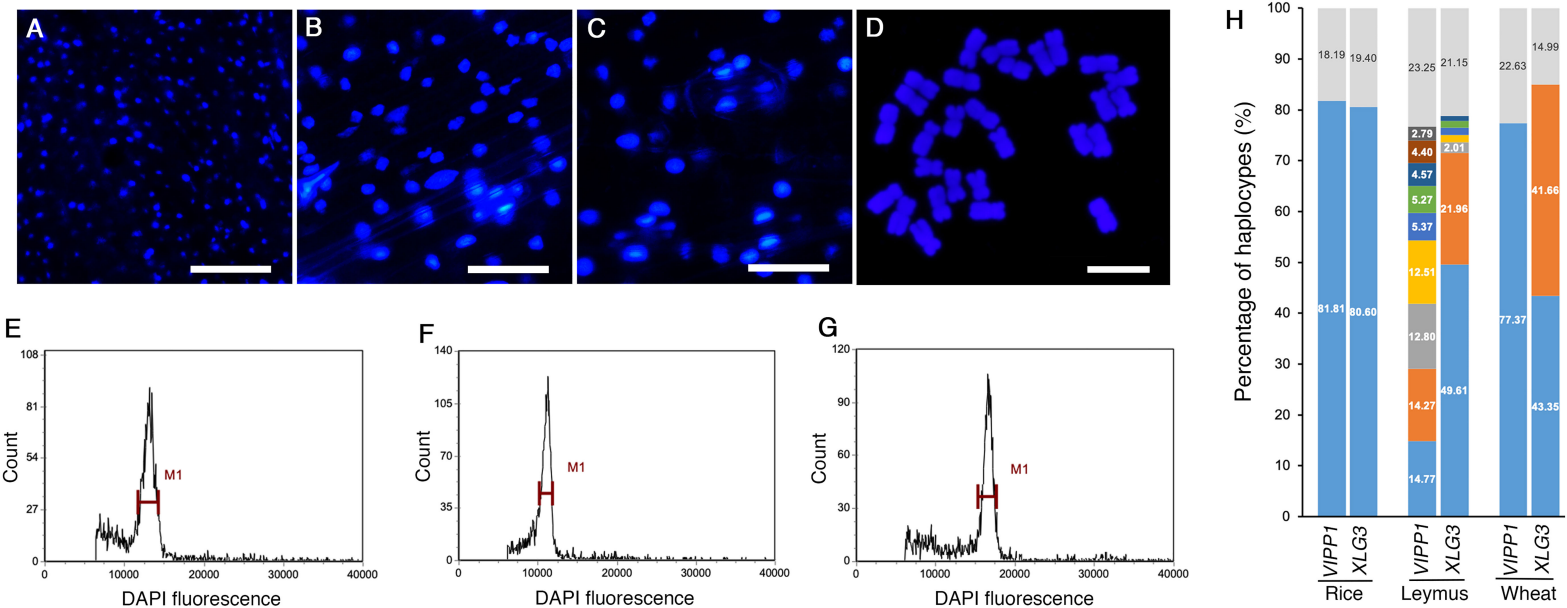
The nucleus size, chromosome ploidy and DNA diversity of different species. Microscopic images of DAPI-stained nuclei of rice **(A)**, Leymus **(B)** and wheat **(C)**. Bars=60 μm. **(D)** Chromosomes of *Leymus chinensis* stained with DAPI. Bar=10 μm. FLC analysis of the nuclei of rice **(E)**, Leymus **(F)** and wheat **(G)**. **(H)** The percentage of haplotypes of *VIPP1* and *XLG3* from different species. The top regions of each gene show the total proportion of haplotypes less than 1.0%. The lower regions represent the haplotype percentages of different genes. The colored regions without numbers indicate 1.52, 1.37, 1.33 and 1.05 for *LeymusXLG3* from the lower to upper parts.

Both sequencing methods verified that many nuclear DNA paralogs coexisted in an individual *Leymus* leaf. For example, if the H9 haplotype of *VIPP1* existed as a single copy in the *Leymus* nucleus, there could be approximately 27 copies of other *VIPP1* variants in the same cell. Similarly, at least 74 *XLG3*, 35 *ACT1*, 86 *NYC1* and 57 *VTE3* paralogs could be found in one nucleus of *Leymus chinensis*. We wondered whether the nucleus size of *Leymus chinensis* could be enlarged by a high level of DNA sequences if all variants coexisted in the same cell. Together with *Leymus chinensis*, two other monocotyledons, rice and wheat, were used to characterize their nucleus size. Photos of DAPI-stained nuclei (**Figures 4A–C**) and statistical analysis (**Supplementary Figure S7A**) both indicated that the nucleus size of *Leymus* was in the medium range and was larger than that of rice and smaller than that of wheat. Chromosome ploidy detection in root tips confirmed that the *Leymus chinensis* used in this study was tetraploid (**Figure 4D**). Compared with diploid rice and hexaploid wheat, it was reasonable that the size of the *Leymus* nucleus was within the normal range. The above data indicated that the high number of DNA paralogs in the *Leymus* nucleus did not increase its size. Generally, tetraploid chromosomes also cannot produce a large number of variants, such as those inside *Leymus* cells. Similar to those of rice and wheat, the FLC analysis of *Leymus* nuclei also presented a single peak (**Figures 4E–G**), which suggested that the nuclear contents in different cells were equivalent in *Leymus* leaves. Nucleotide diversity analysis was carried out with leaves of rice and wheat following the same method as that for *Leymus chinensis*. In addition to wheat *XLG3*, three other DNA fragments were cloned as a single copy (**Figure 4H**; **Supplementary Figure S7B, C**). Based on *VIPP1* and *XLG3* fragments, together with the sequencing results of *ACT1*, *NYC1* and *VTE3*, gene copies in *Leymuschinensis* far exceeded those in rice and wheat. However, the size of the *Leymus* nucleus did not show an increase and remained in the normal range. All of these results suggested another possibility: these variants of unique genes were distributed in different cells in an individual *Leymus* leaf.

### DNA diversity was not found in *VIPP1* and *XLG3* sequences cloned from a single cell of *Leymus chinensis*

**Figure 5:**
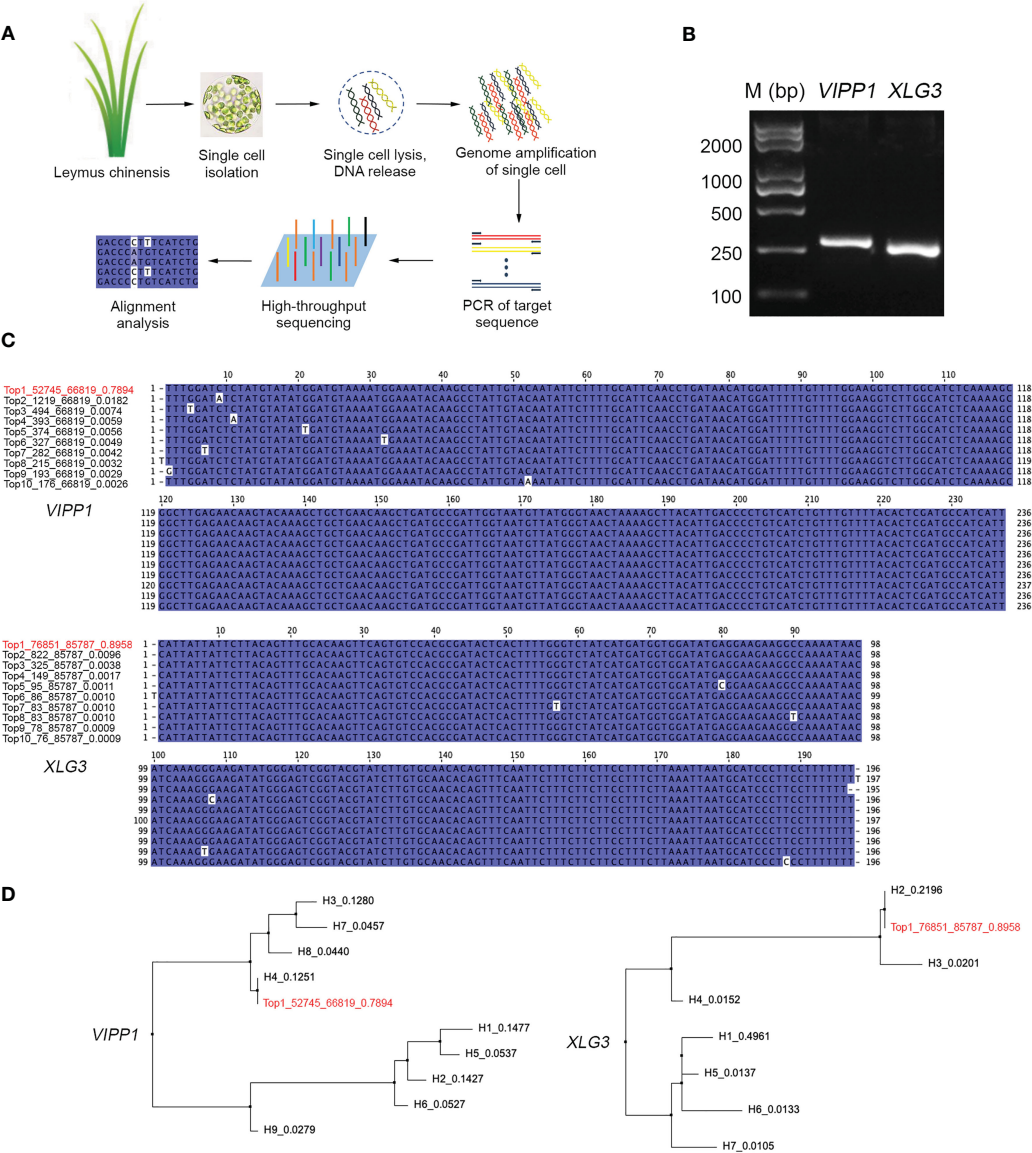
Characterization of *VIPP1* and *XLG3* sequences in a single cell of *Leymus chinensis*. **(A)** The workflow for high-throughput sequencing of unique genes in a single cell of *Leymus chinensis*. **(B)** The PCR products of *VIPP1* and *XLG3* of a single *Leymus* cell. **(C)** Multiple alignment of *VIPP1* and *XLG3* sequences from a single cell of *Leymus chinensis*. ‘Top1_52745_66819_0.7894’ means ‘rank_target reads_total reads_haplotype percentage’. The red color indicates the item with the highest proportion among all reads. **(D)** Comparison of *VIPP1* or *XLG3* sequences from leaf and a single cell. The neighboring-joining trees were set up with the DNA sequences of *VIPP1* and *XLG3* in **Figures 3B, C** plus the top 10 sequences (in red color) from a single cell of *Leymus*.

If DNA polymorphisms in *Leymus chinensis* were inter- but not intracellular variation, the similar mutations of *VIPP1* and *XLG3* fragments observed within leaf tissue could not be detected from a single cell. Here, protoplasts were isolated from *Leymus* leaves, and a single cell was randomly picked for nuclear DNA analysis (**Figure 5A**). A large number of reads for *VIPP1* (66,819) and *XLG3* (85,787) were produced after NGS on the PCR products from the whole-genome amplification of pooled single-cell DNA (**Figure 5B**). The top 10 DNA variants in *VIPP1* and *XLG3* were subjected to multiple alignment analysis. Some sporadic nucleotide mutations were found, but no regular SNP was detected for either gene (**Figure 5C**). In fact, only one kind of *XLG3* ‘Top1_76851_85787_0.8958’ was regarded as a valid sequence because the proportions of other variants were less than 1.0%. In the case of *VIPP1*, even ‘Top2_1219_66819_0.0182’ was over 1.0%, but the single nucleotide mutation (A/C) was only found in this variant and could not be found in other variants at this position, which suggested that this mutation was an error. Only one kind of *VIPP1*, ‘Top1_52745_66819_0.7894’, was cloned in this *Leymus* cell. Clearly, the DNA sequences of *VIPP1* and *XLG3* for a single cell did not show the polymorphism found for leaf tissue, which indicated that nuclear diversity of *Leymus chinensis* occurred between different cells.

Comparing the unique sequences of *VIPP1* or *XLG3* in single cells with their corresponding variants from leaf tissue, ‘Top1_52745_66819_0.7894’ was the same as H4 in leaves, while ‘Top1_76851_85787_0.8958’ was identical to H2 in leaves (**Figure 5D**). The high proportion of *VIPP1* H4 (12.51%) implied that the cells holding this kind of haplotype were prevalent in the leaves, as were cells with *XLG3* H2 (21.96%), which coincided with high probabilities of selection for cells holding *VIPP1* ‘Top1_52745_66819_0.7894’ and *XLG3* ‘Top1_76851_85787_0.8958’.

### Characterization of SNP variants of *Leymus* nuclear DNA

**Figure 6:**
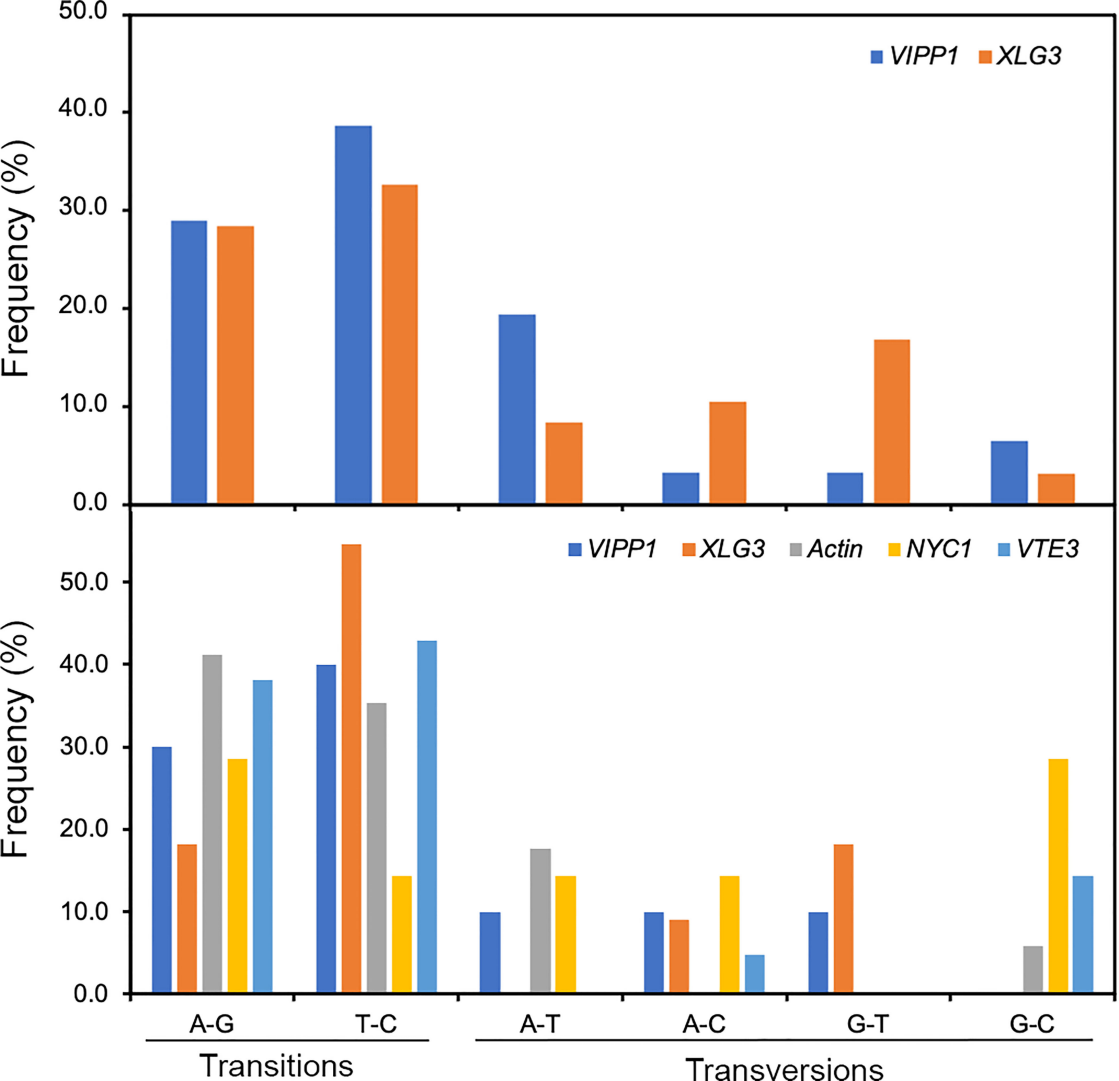
Frequency of SNPs in *VIPP1*, *XLG3*, *ACT1*, *NYC1* and *VTE3* fragments. The different types of SNPs, transitions (A/G and T/C) and transversions (A/T, A/C, G/T and G/C), were counted on the basis of multiple alignment analysis of target genes from Sanger sequencing (upper panel) or from high-throughput sequencing (lower panel). The proportion of each type of SNP out of the total SNPs (not including indels) was calculated.

SNPs are individual nucleotide base differences between different DNA sequences. SNPs are generally biallelic and can be categorized according to nucleotide substitution as either transitions (A/G or T/C), transversions (A/T, A/C, G/T, or G/C) or nucleotide deletions/insertions. In general, for a variation to be considered a true SNP, it must occur in at least 1.0% of the population. Clearly, nucleotide variations among different sequencing reads of *Leymus* nuclear DNA conformed to the features of SNPs. Compared with nucleotide substitutions, DNA deletions/insertions occurred less frequently in cells of *Leymus* leaves and were only detected in introns of longer *XLG3* (10 sites) and *ACT1* (3 sites) fragments (**Figure 2C**; **Supplementary Figure S5B**). Similar to SNPs in other species, nucleotide transitions occurred more frequently than transversions in *Leymus*. **Figure 6** upper panel shows that the transition rate was higher than the transversion rate for both genes, with an average transition/transversion ratio of 2.1 for *VIPP1* and 1.6 for *XLG3*. Similarly, the average frequency of transitions was also higher than that of transversions for shorter DNA sequences sequenced by NGS (**Figure 6**, lower panel). Therefore, the nucleotide variation in *Leymus* cells did not occur randomly but followed a typical SNP pattern.

### DNA polymorphisms in coding regions and their impacts on amino acid sequences

**Figure 7:**
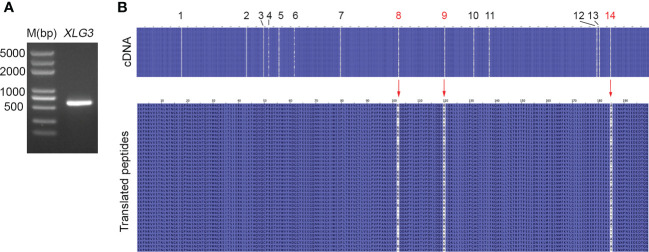
Nucleotide diversity of *XLG3* cDNA. **(A)** PCR product of the *XLG3* cDNA fragment in *Leymus* leaf. **(B)** Multiple sequence alignments of *XLG3* cDNA sequenced with the Sanger method and their corresponding translated peptides. The figures above indicate the SNP sites. Red arrows indicate mutated amino acids resulting from mutated nucleotides marked in red.

Nucleotide variation does not always change the encoded amino acids if nucleotide diversity results from synonymous mutation. **Supplementary Figure S8A** illustrates that all nucleotide variations in *VIPP1* exons were synonymous mutations, and the amino acid sequence remained consistent between different reads. In contrast to *VIPP1*, 3 of 14 SNPs in *XLG3* exons resulted in mutations in the encoded protein (**Supplementary Figure S8B**). Closer inspection showed that these nonsynonymous mutations arose from changes in the first (indicated by a red ‘14’) or second (indicated by a red ‘8’ or ‘9’) nucleotide of a codon (**Table 1**, **Supplementary Figure S8B**). To verify the mutations in *XLG3* exons, which were putatively spliced according to their homology with Aegilops, part of the *XLG3* cDNA corresponding to the spliced exons was cloned and sequenced with the Sanger method. Multiple sequence alignment of *XLG3* cDNA showed the same pattern of nucleotide diversity observed in artificially assembled exons: the positions and patterns of the SNPs in both the spliced exons and cDNA fragments of *XLG3* as well as the residue changes resulting from nucleotide mutations were identical to each other (**Figure 7**). On the basis of the above results, we found that the majority of somatic mutations in GG *Leymus chinensis* nuclei were synonymous and that only a small number of nuclear mutations resulted in changes in the encoded proteins. Further studies were carried out with full-length coding sequences (CDS) of *VTE3* and *NYC1*, which were from GG and YG *Leymus chinensis*, respectively. Both genes showed similar DNA variation as that of *XLG3* cDNA, and numerous SNPs were detected in the whole coding regions. The mutations sites decreased when CDS were translated into their corresponding proteins, which was resulted from synonymous mutations among DNA variations. *AtpC*, a nuclear gene encoding γ subunit of ATPase, does not contain any intron within the reading frames in Aegilops. Sequencing of *AtpC* of YG *Leymus chinensis* also showed a similar DNA polymorphism to those observed in GG ecotype (**Supplementary Figure S8**). All of these data suggested that nuclear DNA variation followed a similar pattern in both ecotypes of *Leymus chinensis*.

**Table 1:** The effects of mutated nucleotides of *XLG3* on its codons and amino acids.

Site	1	2	3	4	5	6	7	8	9	10	11	12	13	14
Codons of exons	ATT ATC	TTG CTG	CAG CAA	CCA CCC	CAC CAT	GAC GAT	TCG TCA	**A**G**A** **A**T**A**	**A**G**A** **A**A**A**	GGA GGG	ATA ATT	TTC TTT	GAG GAA	G**CT** A**CT**
Codons of cDNA	ATT ATC	TTG CTG	CAG CAA	CCA CCC	CAC CAT	GAC GAT	TCG TCA	**A**G**A** **A**T**A**	**A**G**A** **A**A**A**	GGA GGG	ATA ATT	TTC TTT	GAG GAA	G**CT** A**CT**
Amino acid	I	L	Q	P	H	D	S	**R**/I	**A**/K	G	I	F	E	**A**/T

red letter indicated mutated nucleotides and amino acids. The bold letters indicated the mutated amino acids and their corresponding codons.

### Polymorphism of nuclear DNA and its encoded proteins in an individual *Leymus chinensis* seed

**Figure 8:**
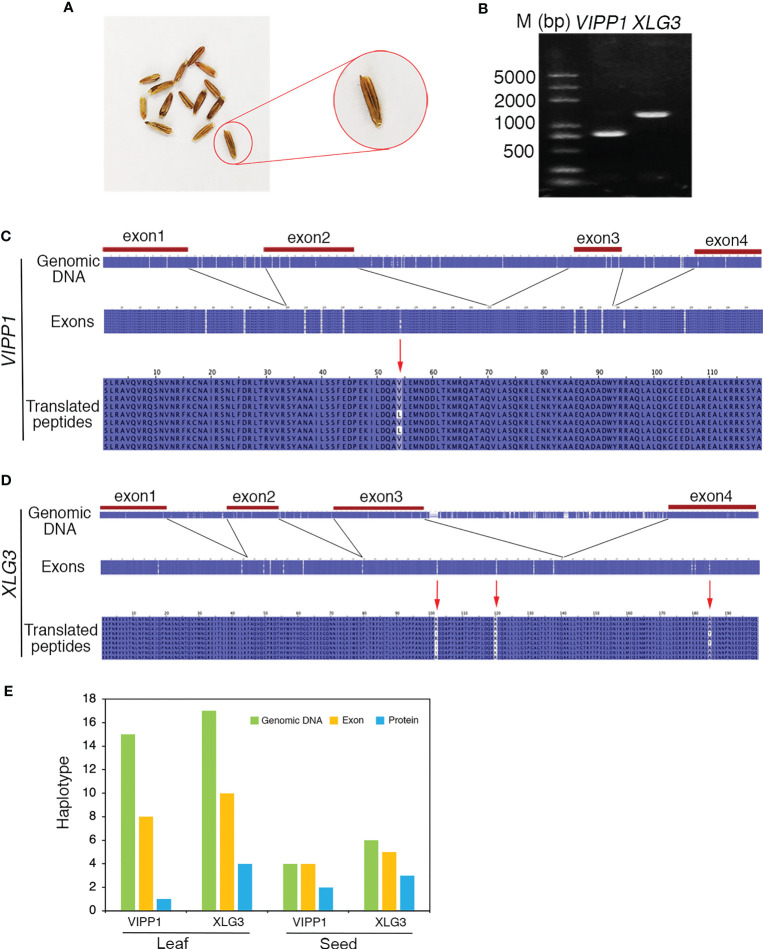
Nucleotide diversity of nuclear genes in one seed of *Leymus chinensis*. **(A)** The phenotype of *Leymus* seeds. **(B)** PCR products of the genomic fragments of *VIPP1* and *XLG3* from one seed of *Leymus chinensis*. **(C)** Multiple alignment of the nucleotide and corresponding amino acid sequences of *VIPP1*. **(D)** Multiple alignment of the nucleotide and corresponding amino acid sequences of *XLG3*. **(E)** The haplotype numbers of genomic DNA, exons and putatively translated peptides of *VIPP1* and *XLG3*.

To clarify whether the somatic mutations in the leaf of *Leymus chinensis* were also present in other organs, such as seeds, a single seed of GG *Leymus chinensis* was randomly chosen and used for total DNA extraction. The same fragments of *VIPP1* and *XLG3* obtained from the leaf were cloned from the seed and sequenced (**Figures 8A, B**). The multiple sequence alignment of 9 independent reads of both genes revealed a similar pattern of nuclear diversity to that found in the leaf (**Figures 2C, D**). The SNP frequency reached 45.7 and 80.1 SNPs per kilobase pair for *VIPP1* and *XLG3*, respectively. In addition, nucleotide variation was again found to be higher in introns than in exons (**Figures 8C, D**). In contrast to the situation in the leaf, a residue change in *VIPP1* was observed in the seed. This mutation arose from SNP p162 in an exon, which was absent from *VIPP1* in the *Leymus chinensis* leaf. The position and pattern of *XLG3* residue variation were consistent between the seed and leaf (**Figures 7B**, **8D**). The above data indicated that the leaf and seed of *Leymus chinensis* shared a similar pattern of nuclear variation in the examined *VIPP1* and *XLG3* fragments and that somatic mutations occurred as early as the seed stage in this grass. In *Leymus* leaves and seeds, the density of SNPs gradually decreased from genomic DNA to its coding amino acids. **Figure 8E** indicates that the number of haplotypes in seeds and leaves decreased gradually from genomic DNA to the corresponding protein, which would be a good way to reduce the differences between *Leymus* cells and enhance their consistency.

## Discussion

In general, gene cloning and DNA sequencing are important first steps in molecular biological research. On the basis of DNA sequencing, downstream experiments, such as protein expression, mutation creation, and evolution analysis, can be carried out. However, when we study *Leymus chinensis* at the molecular level, this regular research method is no longer effective because we cannot obtain a definite DNA sequence from a given pair of primers as numerous different nucleotide sequences are present. In this study, the intraorganismal genetic heterogeneity of *Leymus chinensis* was explored: 1) the nuclear DNA showed genetic heterogeneity among cells, but the chloroplast genes remained consistent; 2) a high density of SNPs was found in the variants of the unique DNA sequences; and 3) the mutation rate decreased from the genomic DNA sequence to the corresponding protein sequence.

Mosaicism, a kind of intercellular genetic heterogeneity, is very difficult to detect within a single organism, although it undoubtedly occurs (Otto and Hastings, 1998; Lei and Jan, 2018). A mutation rate of 2.7×10^-8^ per base pair per generation has been recognized as the highest intraorganismal genetic heterogeneity rate in eukaryotes (Hanlon et al., 2019), which implies that the overwhelming majority of cells in the mosaic organism remain homogeneous. In this study, different species, different organs, different DNA segments, different sequencing methods and even a single cell were employed to characterize nucleotide mutations in *Leymus chinensis*, conclusively suggesting that DNA diversity occurred among different cells. In other words, the majority of *Leymus* cells are genetically heterogeneous. The number of haplotypes increased with the extension of DNA sequencing (**Figures 3D, E**). Therefore, it is reasonable that more haplotypes would be discovered if longer segments of *ACT1*, *NYC1* and *VTE3* were sequenced. Given the similar level of genetic diversity between other nuclear genes as those studied here, the total number of haplotypes at the level of the *Leymus chinensis* genome could be extremely high. *Leymus chinensis* has previously been found to be an allopolyploid species (2n=4x=28, NsNsXmXm) (Yen et al., 2009), which was confirmed in this study by chromosome counting from root tip cells (**Figure 4D**). Hexaploid wheat (*Triticum aestivum*, genomes AABBDD) was used as control to detect DNA polymorphism in this polyploid species. Both number and proportions of different haplotypes of different DNA fragments (**Figure 4H**; **Supplementary Figure S5**) in *Leymus chinensis* has showed totally different characters. In addition, the DNA sequences of *VIPP1* and *XLG3* for a single cell did not show the polymorphism found in leaf tissue (**Figure 5**). All of these results suggested that these variants of unique genes should be distributed in different cells of *Leymus chinensis*. There are two ecotypes in the species categorized according to leaf color, gray green and yellow green types. In the wild, patches of the two ecotypes were always mosaic distribution (Yuan et al., 2016). Both genomic DNA (*AtpC*) and cDNA (*NYC1*) of YG *Leymus chinensis* showed similar pattern of DNA variation (**Supplementary Figure S9**). Not only in *Leymus chinensis*, DNA polymorphism was also detected in its relative species, *Leymus multicaulis*. Different with those of *Leymus chinensis*, the number of haplotypes of *Leymus multicaulis* homologic genes decreased, indicating DNA diversity of this species was relatively moderate (**Supplementary Figure S6**). All *Leymus* species contain two basic Ns and Xm genomes, with Ns derived from *Psathyrostachys*, whereas the ancestor of Xm remains unclear (Zhang and Dvořák, 1991; Wang et al., 1994). Even *Leymus chinensis* and *Leymus muticaulis* are two representative tetraploid species, it was found that phylogenetic relationships between *Leymus chinensis* and the other *Leymus* species was remote, while this grass was close to *Hordeum bogdanii* (Yang et al., 2012). It was reasonable that DNA diversity of *Leymus muticaulis* was different with that of *Leymus chinensis* and less intensive, but it occurred definitely in this species.

In contrast to the examined nuclear genes, the sequences of *psbA* and *ndhH* remained consistent in the leaves of *Leymus chinensis* (**Figure 2D**). Genes within the nucleus evolve faster than those in chloroplasts (Wolfe et al., 1987), which is related to the slower mutation rate in the chloroplast, possibly owing to differences in replication fidelity or rates of mismatch repair (Ono et al., 2007; Morley and Nielsen, 2016). Although we cannot exclude the possibility of mutations in other chloroplast genes, we tentatively infer that the chloroplasts of *Leymus chinensis* exhibit a greater ability to maintain genetic homogeneity.

SNPs have been developed as genetic markers to distinguish individuals among the same or different species. In this research, ‘the individual’ does not refer to a single *Leymus* seedling but a single cell because SNPs occurred intercellularly. The DNA sequences of different cells of an individual *Leymus* leaf varied in their SNPs (**Figure 2**). These genetically heterogeneous cells of the leaf or seed actually originated from a single cell—the zygote. The DNA mutation of *Leymus* possibly occurred in the process of cell proliferation accompanied by DNA replication. DNA replication during cell division has been hypothesized to be a leading cause of genetic mutation (Baer et al., 2007; Gaut et al., 2011). The similar SNP profile shared between the leaf and seed suggested that nucleotide mutations found in *Leymus chinensis* follow a certain rule and are not random.

The accumulation of somatic mutations has been proposed to cause a series of problems, including diseases and death, in an individual organism (Dubrovina and Kiselev, 2016). *Leymus chinensis* obviously represents an exception to this pattern, as it can grow normally in both the field and the laboratory. Leaf anatomical observations suggested that the growth and development of *Leymus* cells were not disturbed by the genetic diversity among them (**Supplementary Figure S10**). This may have been related to the lower level of protein variation relative to the high level of nuclear DNA variation. Somatic diversity gradually decreased from the level of genomic DNA to exons to proteins in both the seed and leaf of *Leymus chinensis* (**Figure 8E**). It has been reported that coding regions are more conserved than intergenic regions, promoters and UTRs (Tatarinova et al., 2016). In this study, the SNP abundance in introns was higher than that in exons, which is consistent with evolutionary constraints on the mutability of protein-coding regions (Gelfman et al., 2012). Synonymous mutations in exons are also a strategy for mutation purification. The ratio of nonsynonymous to synonymous nucleotide substitutions (dN/dS) has been used to indicate the ‘purifying selection’ of mutated nucleotides in previous studies (Yu et al., 2020). *Leymus chinensis* clearly shows strong ‘purifying selection’ to constrain the variation in proteins.

## Data availability statement

The data presented in the study are deposited in the GenBank repository, accession numbers are listed in **Supplementary Table S3**.

## Author contributions

HY carried out genes cloning and multiple alignment analysis of the single cell and chloroplast. LM performed nuclear gene cloning and multiple alignment analysis. YZ studied nuclear size of different species. GN and HW collected the *Leymus* seeds and grew them. YS analyzed data of NGS. LW detected chromosome ploidy of *Leymus* root. LZ conceived these studies and finished the manuscript. All authors contributed to the article and approved the submitted version.
