# Supplementary material for: Characterization of nuclear DNA diversity in an individual Leymus chinensis
Source: Front Plant Sci. 2023 Jun 6;14:1157145. doi: 10.3389/fpls.2023.1157145 (PMC10280068; doi:10.3389/fpls.2023.1157145)
Supplement: Supplementary file 1 [file DataSheet_1.pdf]

Supplementary Material

Characterization of Nuclear DNA Diversity in an Individual *Leymus chinensis*

Haoyang Yu[1], Lijuan Ma[1], Ye Zhao, Gaowa Naren, Haiyan Wu, Yongwei Sun, Lei Wu, Lingang Zhang[*]

1 These authors contributed equally to this work.
*** Corresponding Author:** Lingang Zhang
Email address: lingangzhang@imu.edu.cn

This section includes:
Supplementary Figures S1 to S10
Supplementary Tables S1 to S3

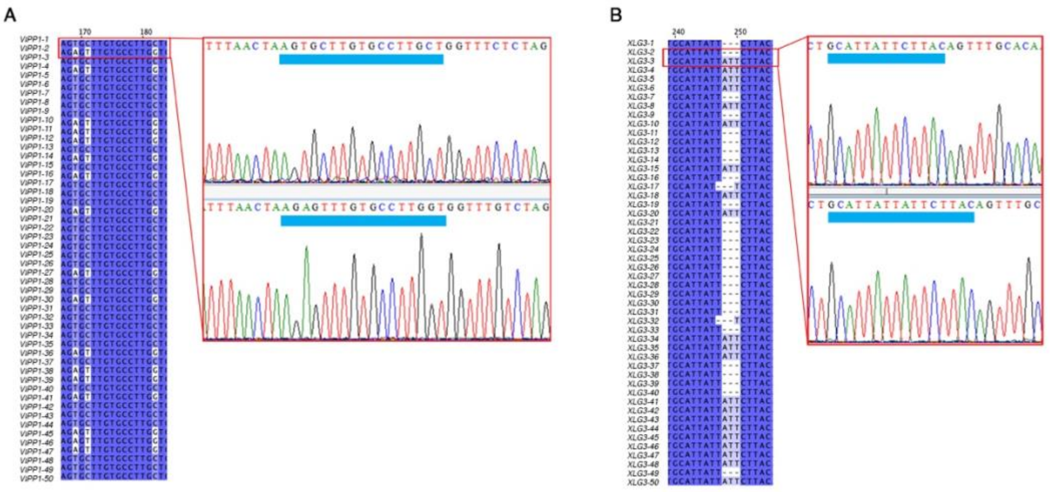

Supplementary Figure S1. Representative sequencing chromatograms of *VIPP1* (A) and *XLG3* (B). Short regions including nucleotide mutations/deletions were randomly chosen from the alignment analysis of *VIPP1* and *XLG3* of FIG.2*C*. The numbers above the aligned sequences indicate nucleotide positions. The red rectangles mark the sequences selected as example chromatogram sequences that were labeled with blue boxes.

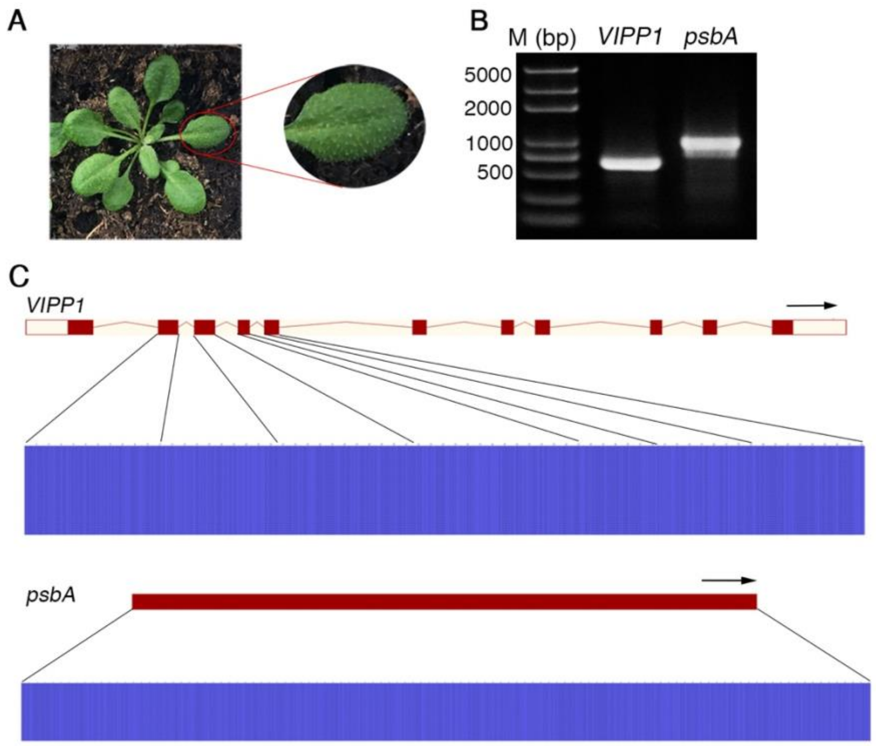

Supplementary Figure S2. Alignment analysis of 50 individual reads of nuclear (*VIPP1*) and chloroplast genes (*psbA*) from a single leaf of *Arabidopsis thaliana*. (A) A four-week-old *Arabidopsis* plant and an enlarged image of a piece of its leaf. **(B)** PCR products of *VIPP1* and *psbA*. The genomic DNA fragments of nuclear (*VIPP1*) and chloroplast genes (*psbA*) were cloned from the same piece of *Arabidopsis* leaf with high-fidelity DNA polymerase. **(C)** Multiple sequence alignments of 50 individual reads of *VIPP1* and *psbA*. Nucleotides were colored for percent identity using Jalview software. The transcript diagrams of different genes are provided above the corresponding genes. Filled boxes represent coding sequences, and unfilled boxes represent UTRs (untranslated regions). Lines connecting the boxes represent introns. Both exons and introns of the DNA sequences are marked according to the transcript diagram. The black arrow indicates the direction of the forward-strand coding sequence.

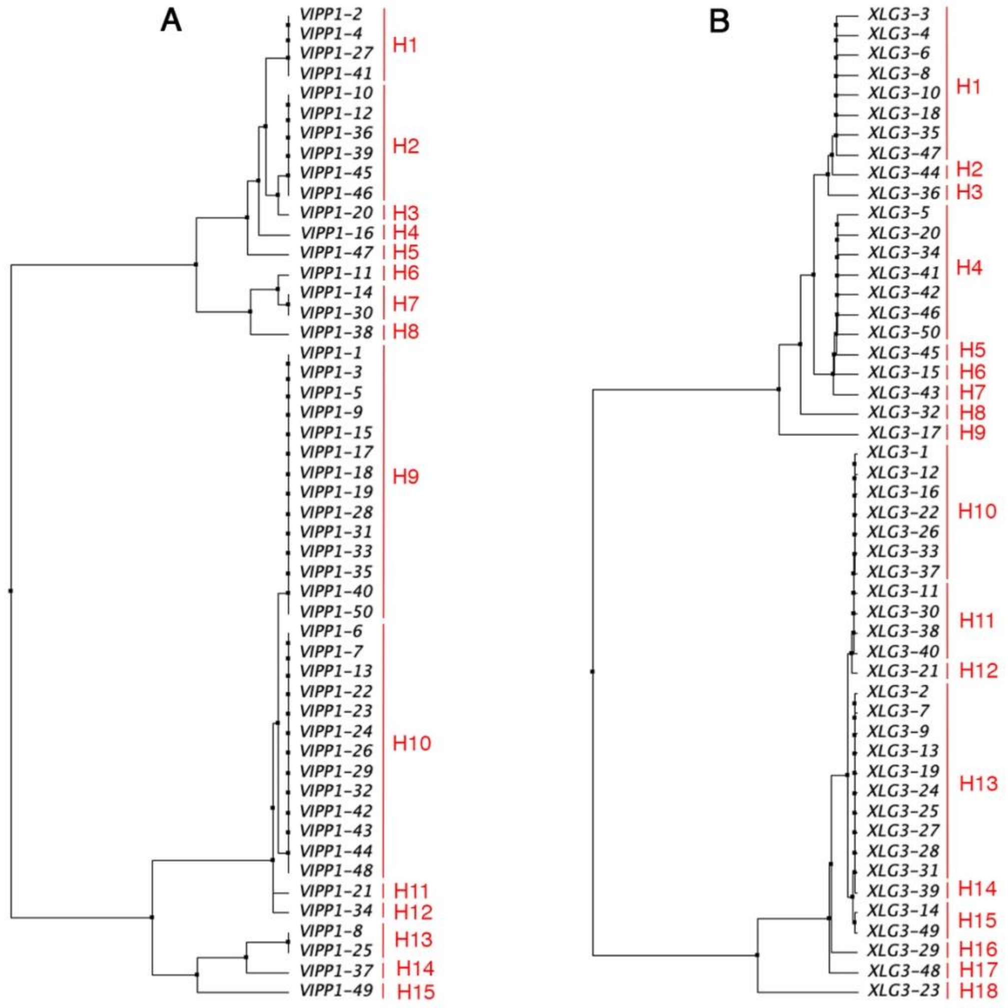

Supplementary Figure S3. Average-distance tree of *VIPP1* and *XLG3* sequences in the *Leymus* leaf. The DNA sequencing reads of *VIPP1* **(A)** or *XLG3* **(B)** were clustered on the basis of the divergence among them. The red 'H' refers to the 'Haplotype'.

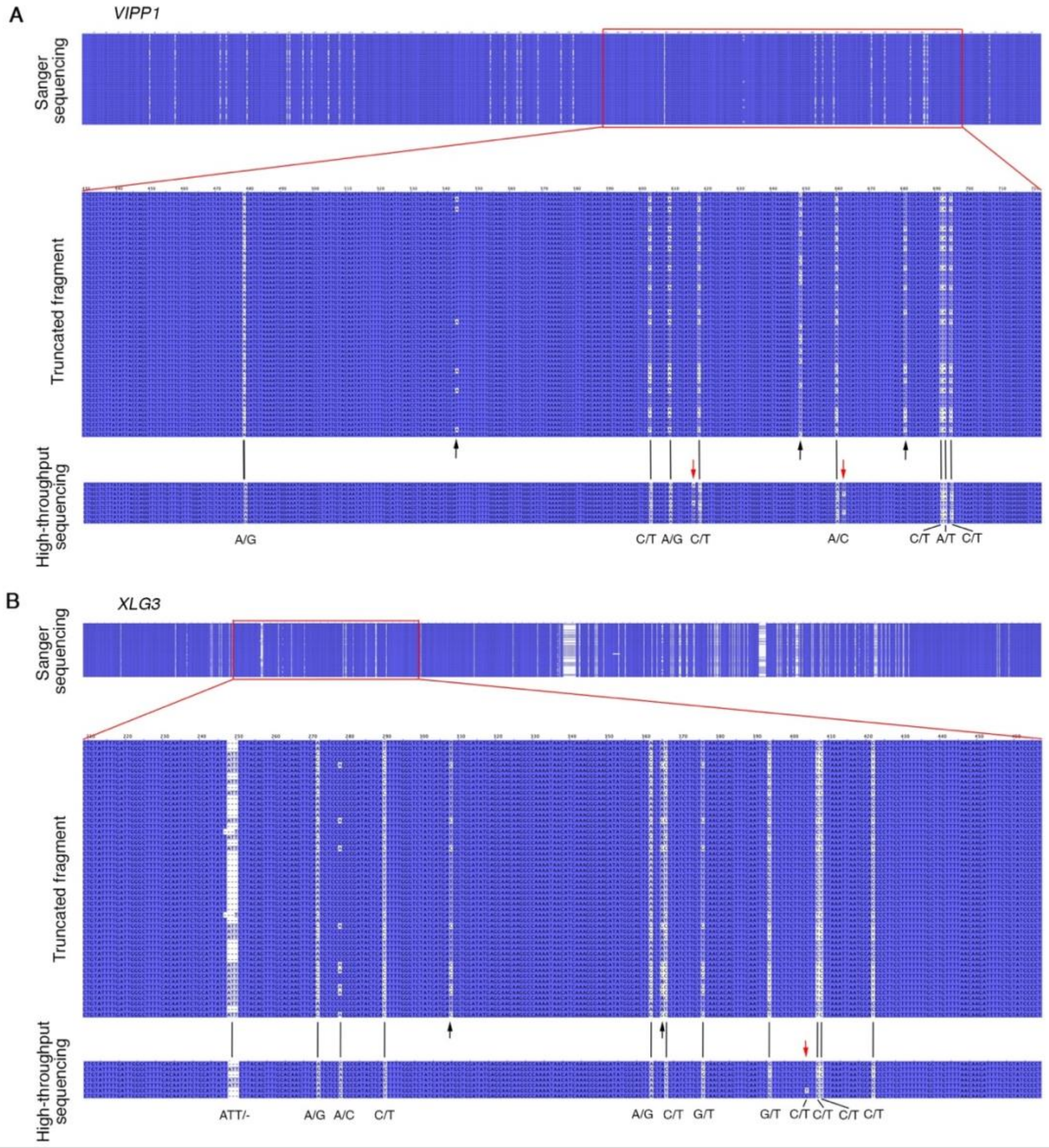

Supplementary Figure S4. Comparison of DNA diversity resulting from different sequencing methods. The red rectangle highlights the truncated fragments of longer *VIPP1* **(A)** or *XLG3* **(B)** sequence reads resulting from the Sanger method. The corresponding shorter sequences of *VIPP1* or *XLG3* resulted from directional PCR amplification and were sequenced with a high-throughput method. The unique sequences (haplotypes) with a proportion over 1.0% were arranged from high to low and aligned with ClustalWS software. The black slash indicates the identical mutation sites, and nucleotide mutations are marked below. The black arrow indicates mutations that were only present in the Sanger method results. The red arrows indicate mutations that were only present in the high-throughput method results.

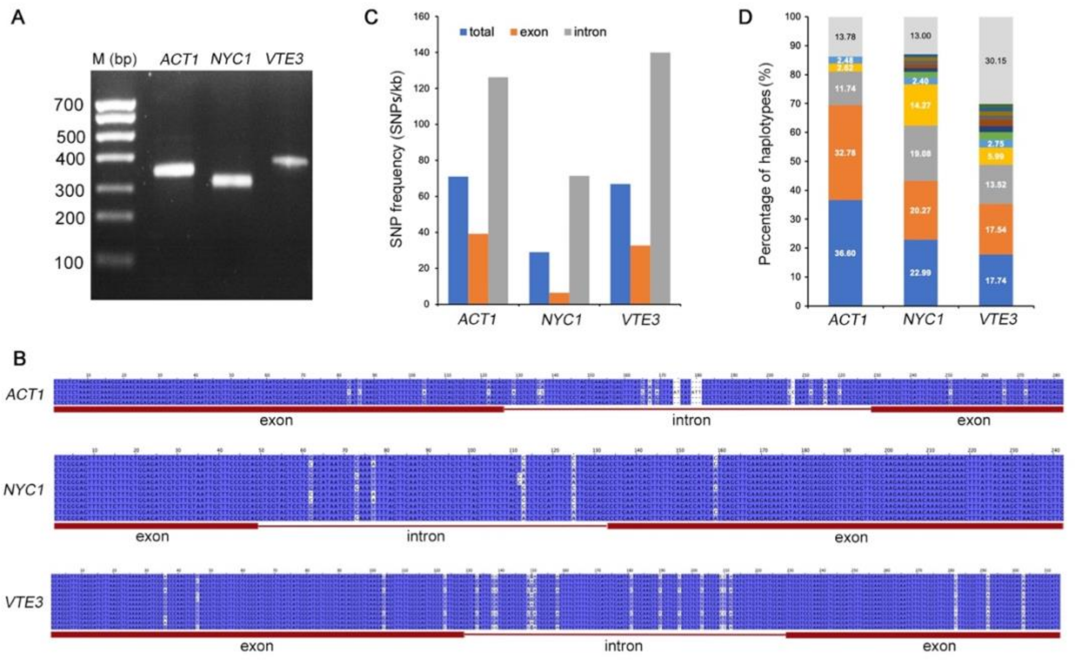

Supplementary Figure S5. Characterization of nucleotide diversity of *ACT1*, *NYC1* and *VTE3* of *Leymus chinensis*. (A) PCR products of *ACT1*, *NYC1* and *VTE3*. **(B)** Multiple sequence alignments of *ACT1*, *NYC1* and *VTE3*. After high-throughput sequencing, the unique sequences with a proportion over 1.0% were arranged from high to low. Nucleotides were colored for percentage identity using Jalview software. The 'exon' and 'intron' regions are marked below. **(C)** Statistics of SNP abundance of these three genes. **(D)** The percentage of haplotypes of different genes. The top region (gray) of each gene shows the total proportion of haplotypes less than 1.0%. The lower areas represent the haplotype percentage of different genes. The colored regions (from lower to upper) without numbers indicate 1.97, 1.33, 1.28, 1.27, 1.13, and 1.01 for *NYC1* and 2.51, 2.24, 2.17, 1.52, 1.34, 1.30, and 1.23 for *VTE3*.

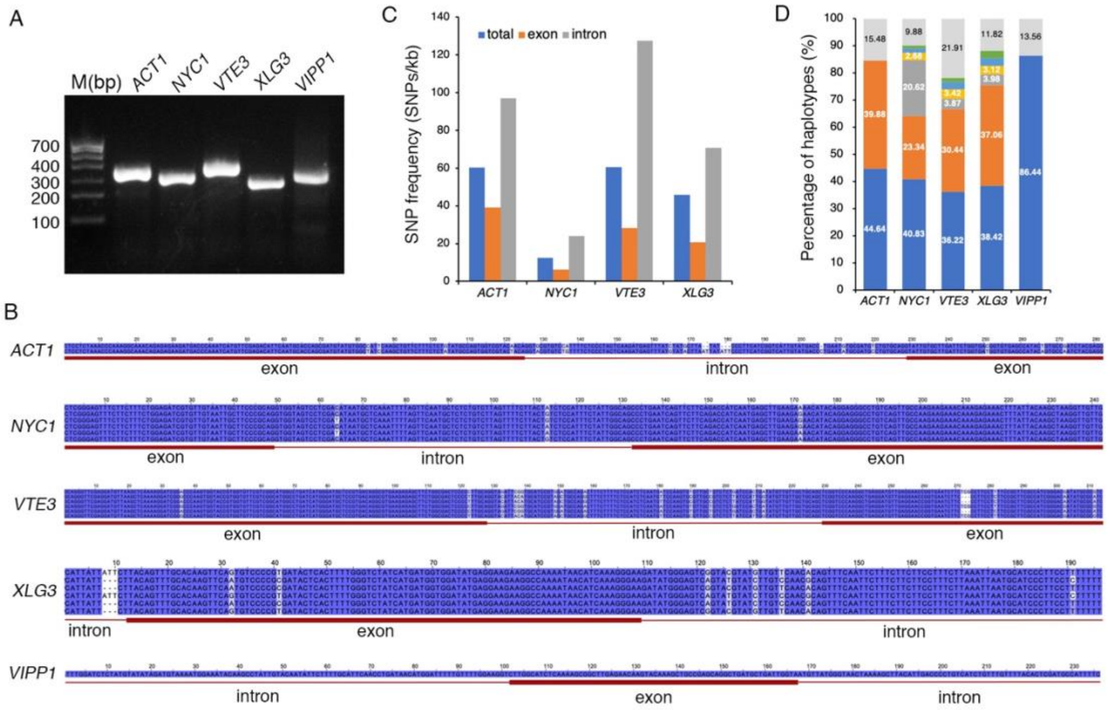

Supplementary Figure S6. Characterization of nucleotide diversity of *ACT1, NYC1, VTE3, XLG3* and *VIPP1* of *Leymus multicaulis*. (A) PCR products of *ACT1*, *NYC1*, *VTE3*, *XLG3* and *VIPP1* of *Leymus multicaulis*. **(B)** Multiple sequence alignments of *ACT1*, *NYC1*, *VTE3*, *XLG3* and *VIPP1* of *Leymus multicaulis*. After high-throughput sequencing, the unique sequences with a proportion over 1.0% were arranged from high to low. Nucleotides were colored for percentage identity using Jalview software. The 'exon' and 'intron' regions are marked below. **(C)** The density of SNP sites in different regions of genes. **(D)** The percentage of haplotypes of different genes. The top region (gray) of each gene shows the total proportion of haplotypes less than 1.0%. The lower areas represent the haplotype percentage of different genes. The colored regions (from lower to upper) without numbers indicate 1.52 and 1.13 for *NYC1*, 2.69 and 1.45 for *VTE3*, 2.87 and 2.73 for *XLG3*.

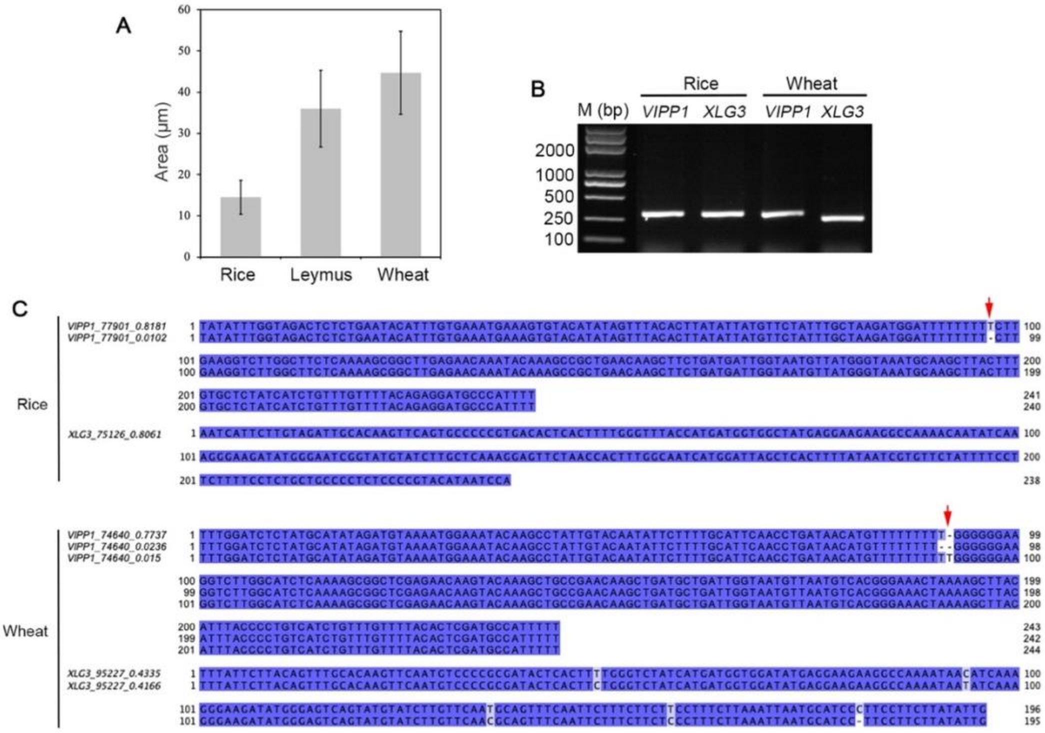

Supplementary Figure S7. The nucleus size and DNA mutation of *VIPP1* and *XLG3* from different species. (A) The statistics of the nucleus size of rice, *Leymus* and wheat. Thirty nuclei stained by DAPI were randomly selected to calculate the area of each nucleus from different species. **(B)** The PCR products of *VIPP1* and *XLG3* from rice and wheat. **(C)** The unique sequences with a proportion over 1.0% were aligned. The nucleotides were colored for percentage identity using Jalview software. Red arrows indicate the mutated sites. ‘*VIPP1*_77901_0.8181’ means ‘gene_total clean reads_haplotype percentage’.

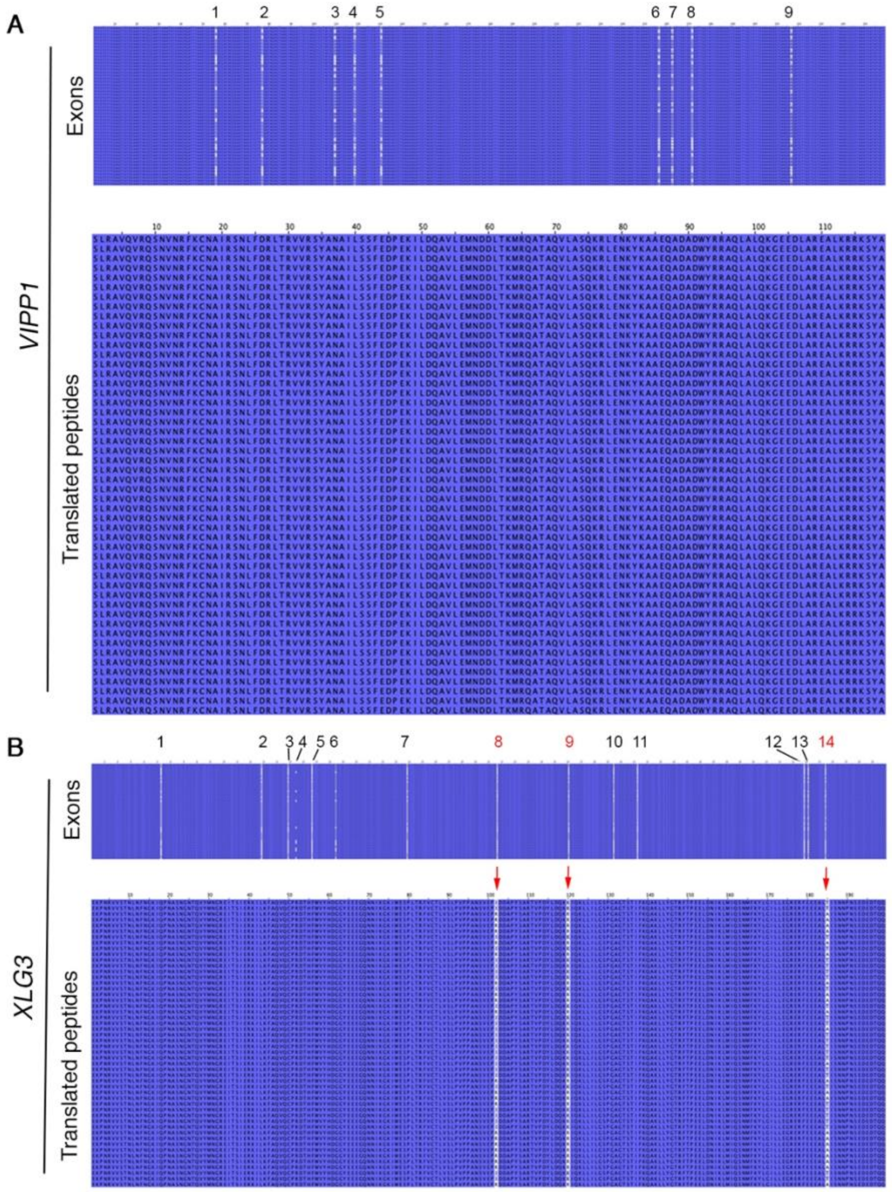

Supplementary Figure S8. Nucleotide mutations in exons of *VIPP1* and *XLG3* and their effects on amino acids. (A) Single nucleotide polymorphism of *VIPP1* exons and their corresponding translated peptides. **(B)** Single nucleotide polymorphism of *XLG3* exons and their corresponding translated peptides. The red numbers mark the sites of SNPs that could induce mutations in amino acids.

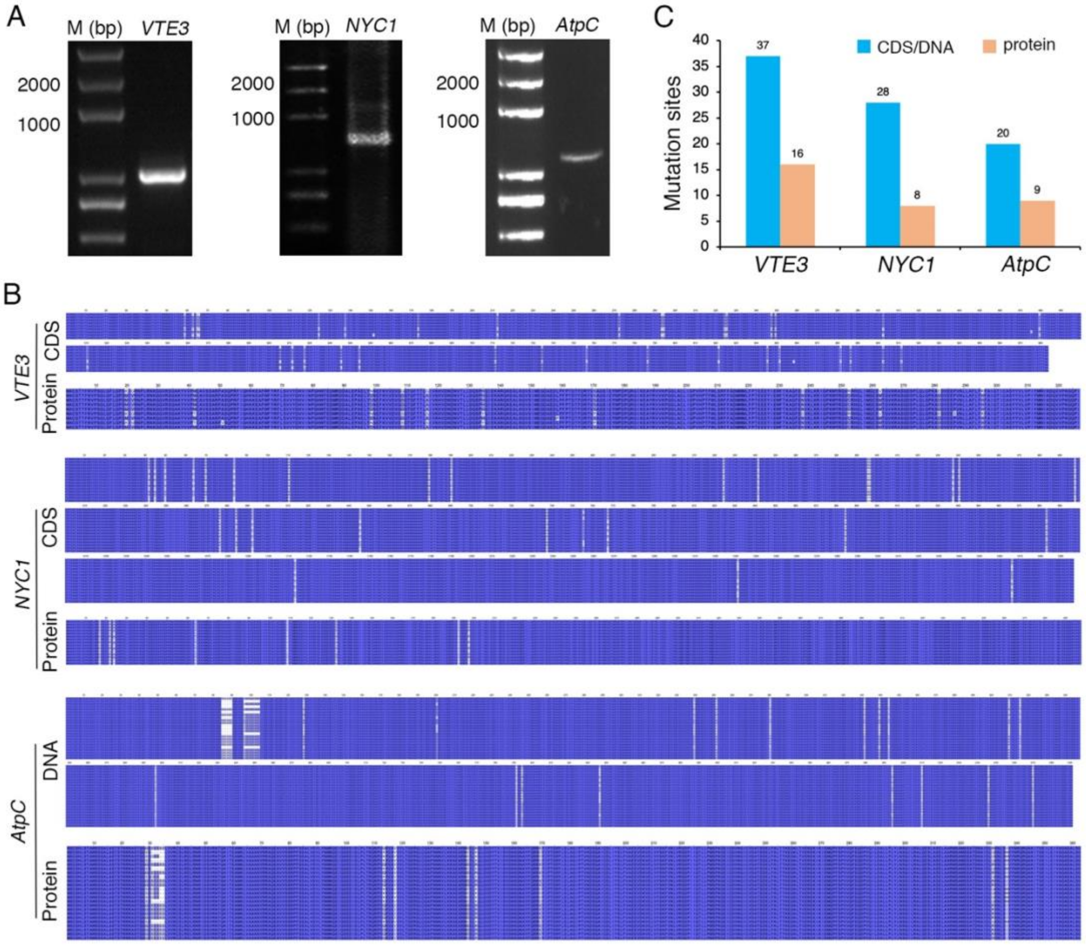

Supplementary Figure S9. Nuclear DNA variation in an individual leaf of different ecotypes of *Leymus chinensis*. (A) PCR products of full-length CDS of *VTE3* (grey green *Leymus chinenesis*) and *NYC1* (yellow green *Leymus chinenesis*), and genomic DNA of *AtpC* (yellow green *Leymus chinensis*). **(B)** Multiple sequence alignments of sequenced reads of above PCR products and their encoded proteins. There are no introns in genomic DNA sequences of *AtpC* of yellow green *Leymus chinensis*. **(C)** The number of mutation sites of CDS/DNA sequences and their corresponding proteins of *VTE3*, *NYC1* and *AtpC*.

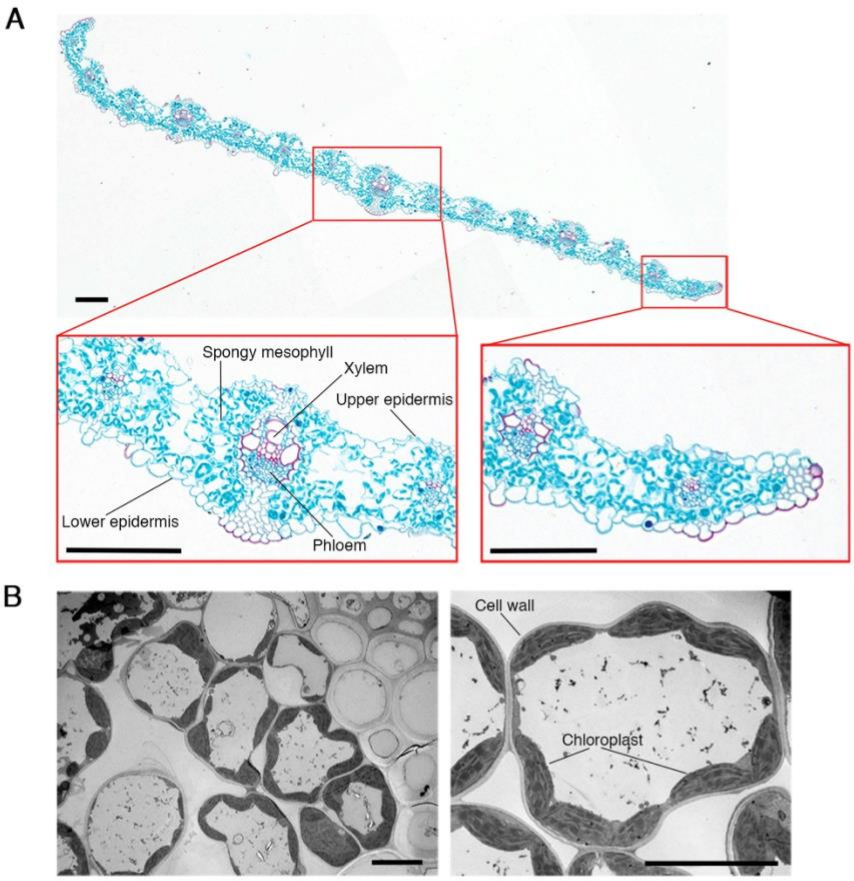

Supplementary Figure S10. The anatomical structure of *Leymus* leaves. (A) Representative photograph of leaf cross-sections stained with Alcian blue and safranin-O. The upper image is a whole cross-section of the leaf. Lower pictures are enlarged images of selected regions. Different tissues are marked in the enlarged image. Bars=100 μm. **(B)** Transmission electron microscopy (TEM) of transverse leaf sections. Bars=10 μm.

Supplementary Table S1. Nuclear DNA diversity of an individual leaf of *Leymus chinensis*

Gene	Region	Aligned sequence length (bp)	SNPs			SNP frequency (SNPs/Kb)	Haplotype
			Transitions	Transversions	Indels		
VIPP1	Exon	359	7	2	0	25.1	8
	Intron	429	14	8	0	51.3	10
	Total	788	21	10	0	39.3	15
XLG3	Exon	600	11	3	0	23.3	10
	Intron	712	47	34/1*	10	127.8	15
	Total	1312	58	37/1*	10	80.0	18

*1: triple mutation A/C/T at position of 605 in *XLG3* intron.

Supplementary Table S2. Primers used in this study

Species	Gene	Primer (5'-3')
Leymus chinensis/ Leymus multicaulis	*VIPP1* (long)	Forward: TTTCTCTTCGAGCTGTGCAAGTACG
		Reverse: AGCATATGATTTACGCCGTTTAAGGGC
	VIPP1 (short)	Forward: CTGCCTCTTATATTACCAGGTTCTTGTTTG
		Reverse: TTGAGCCCTCCGATACCTACATTTAAG
	XLG3 (long)	Forward: GGGAAAAGCCAAACAGGGTTGTC
		Reverse: CTTGTTGTGTATCCTCATCCTCTGAGTTTG
	XLG3 (short)	Forward: CTGTATTTTGATTGGGCTTTTCACAATATCTG
		Reverse: AGGGGATACAGAAAATCTTGTTAAAACAAGC
	ACT1	Forward: GAAGAACATCCAGTTTTGCTGACAGAAG
		Reverse: CGGAGAATAGCATGAGGAAGTGCATATC
	NYC1 (long)	Forward: ATGGCCGCCGCGGTCGTCCACCTC
		Reverse: TTATGTGCCAGGGAAAGGTCCGCC
	NYC1 (short)	Forward: CACAAGAGGGTTAGGAAAAGCACTTG
		Reverse: TCTGGTTTGCAAACATCACAAGATGTAC
	VTE3 (long)	Forward: ATGGCTTGTTCAACCACATTTAGGGTTCC
		Reverse: TTAAATAGGCTGGCCTTTGGGAACAACC
	VTE3 (short)	Forward: AGAAGAGTACATAGAGTGGTTCACCAAG
		Reverse: CATGTAGATAGGCACTAGCACGTAGTAC
	AtpC	Forward: ATGTCGTGCTCCCACCTCTCCAC
		Reverse: TCAGCCGGACAGGGCCTC
	psbA	Forward: TTATCCATTAATAGATGGAACTTCAACAGCAGC
		Reverse: ATGACTGCAATTTTAGAGAGACGCGAAAG
	ndhH	Forward: GAATGGAAAAAATTGCAGAAAACCGAGC
		Reverse: CAAGGAAAACCGCTTTGATCTCCTATG
Rice	*VIPP1*	Forward: GGCCACCTGATTCTTATATTTCCATATTCTTG
		Reverse: TTGAGCCCTCCGATACCTATGATTTCAAG
	XLG3	Forward: CTATCACTATCACTGAATTTGCCTCAATATCTG
		Reverse: ATAAATTACAAAGAGGGAAGGGAGAATGCAC
Wheat	*VIPP1*	Forward: CTGCCTCTTGTATTGTCAGGTTGTTTG
		Reverse: TTGAGCCCTCCGGTACCTACATTTAAG
	XLG3	Forward: CTGTATTTTTATTGGGCTTTTCACAATATCTGC
		Reverse: AGGGGATACAGACAATCAAGTTAAAACAAG
Arabidopsis thaliana	*VIPP1*	Forward: GTGGAGCGCTGAGAGTGAATGTGTTGAGATTAGC
		Reverse: AGCAAAAGACTTTCGTCGTTTAAGGGCCTCACG
	psbA	Forward: ATGACTGCAATTTTAGAGAGACGCGAAAGC
		Reverse: ATTATCCATTTGTAGATGGAGCCTCAACAGC

Supplementary Table S3. GenBank accession numbers of different DNA fragments cloned in this study

Number	Gene Name	GenBank accession number	Description
1	*VIPP1*	OQ473502	Figure 2C, long *VIPP1* of leaf
2	*XLG3*	OQ473503	Figure 2C, long *XLG3* of leaf
3	*ndhH*	OQ473504	Figure 2D
4	*psbA*	OQ473505	Figure 2D
5	*VIPP1*	OQ473506	Figure 8C, long *VIPP1* of seed
6	*XLG3*	OQ473507	Figure 8D, long *XLG3* of seed
7	*XLG3*	OQ473508	Figure 7B, cDNA of leaf *VIPP1*
8	*VIPP1*	OQ473509	Figure 3B, short *VIPP1* of leaf
9	*XLG3*	OQ473510	Figure 3C, short *XLG3* of leaf
10	*VIPP1*	OQ473511	Figure 5C, short *VIPP1* of single cell
11	*XLG3*	OQ473512	Figure 5C, short *XLG3* of single cell
12	*ACT1*	OQ473513	Supplementary Figure S5B
13	*NYC1*	OQ473514	Supplementary Figure S5B
14	*VTE3*	OQ473515	Supplementary Figure S5B
15	*VIPP1*	OQ473516	Supplementary Figure S6C, *VIPP1* of rice
16	*XLG3*	OQ473517	Supplementary Figure S6C, *XLG3* of rice
17	*VIPP1*	OQ473518	Supplementary Figure S6C, *VIPP1* of wheat
18	*XLG3*	OQ473519	Supplementary Figure S6C, *XLG3* of wheat

Note: In this study, different kinds of DNA fragments have been sequenced with Sanger or High-throughput sequencing method. In the case of Sanger sequencing (OQ473502- OQ473508), the first read of different DNA variations has been used for gene record in GenBank. In the case of High-throughput sequencing (OQ473509- OQ473519), the DNA read of the highest-proportion haplotype was used for gene record in GenBank.
